# Free surface equatorial flows in spherical coordinates with discontinuous stratification depending on depth and latitude

**DOI:** 10.1007/s10231-022-01214-w

**Published:** 2022-04-28

**Authors:** Calin Martin, Adrian Petruşel

**Affiliations:** 1 Faculty of Mathematics, University of Vienna, Oskar-Morgenstern-Platz 1, 1090 Vienna, Austria; 2 Faculty of Mathematics and Informatics, Babeş-Bolyai University, Cluj-Napoca, Romania; 3 Academy of Romanian Scientists, Bucharest, Romania

**Keywords:** Azimuthal flows, Discontinuous density, Spherical coordinates, Coriolis force, Implicit function theorem, 35Q31, 35Q35, 35Q86, 35R35, 76E20

## Abstract

We derive and subsequently analyze an exact solution of the geophysical fluid dynamics equations which describes equatorial flows (in spherical coordinates) and has a discontinuous fluid stratification that varies with both depth and latitude. More precisely, this solution represents a steady, purely–azimuthal equatorial two-layer flow with an associated free-surface and a discontinuous distribution of the density which gives rise to an interface separating the two fluid regions. While the velocity field and the pressure are given by means of explicit formulas, the shape of the free surface and of the interface are given in implicit form: indeed we demonstrate that there is a well-defined relationship between the imposed pressure at the free-surface and the resulting distortion of the surface’s shape. Moreover, imposing the continuity of the pressure along the interface generates an equation that describes (implicitly) the shape of the interface. We also provide a regularity result for the interface defining function under certain assumptions on the velocity field.

## Introduction

Flow stratification represents a pronounced feature of geophysical ocean flows that particularly applies to large scale ocean movements [17, 29]. To exemplify the previous aspect we point out the existence of a band of 150 km on each side of the Equator, stretching out longitudinally over about 16.000 km throughout the extent of the Pacific Ocean, where the stratification gives rise to a sharp interface (called pycnocline of thermocline) [16]. The thermocline separates a shallow near-surface layer of relatively warm water from a deep layer of colder and denser water.

Another important aspect that one needs to consider in the investigation of geophysical ocean flows refers to the effects generated by the presence of Coriolis forces–created by the Earth’s rotation. Indeed, together with the prevailing westward wind pattern, Coriolis forces generate an underlying current field that presents flow-reversal [2, 12]: in a band of about $2^{\circ }$ latitude around the Pacific Equator the current field shifts from a westward flow near the surface to an eastward flowing jet called the Equatorial Undercurrent (EUC) whose core resides approximately on the thermocline.

The interaction of currents and (surface and internal) waves which also takes into account the density stratification is a recent avenue of research [5–7, 11, 27] performed within the setting of nonlinear geophysical governing equations. We find important to mention that the issue of stratification was considered from a rigorous analytical perspective only (relatively) recently: a selective list of works dealing with two-dimensional gravity water flows (without Coriolis effects) consists of [13, 14, 18, 20, 35–37].

Rigorous mathematical analyses of the aspects mentioned earlier were started by Constantin and Constantin & Johnson who constructed exact solutions exhibiting various features shared by geophysical ocean flows, like three-dimensionality [4, 10, 26], equatorially-trapped waves [3], presence of underlying currents [19], or a preferred propagation direction, cf. [8, 9], where an approach that takes into account the spherical shape of the Earth was initiated. The latter approach (by means of spherical coordinates) was extended by Henry & Martin [21–24] to construct exact azimuthal equatorial flows allowing for continuous stratification (depending on depth and latitude) and by Martin & Quirchmair [30] to devise exact continuously stratified solutions concerning the Antarctic Circumpolar Current (ACC). Moreover, exact azimuthal solutions with a discontinuous density stratification, giving rise to internal waves and pertaining to EUC and ACC were presented recently by Martin [31] and Martin & Quirchmair [32], respectively.

In this paper we broaden the choice for the density function from [31] to the case of a discontinuous density that varies with both depth and latitude. The relevance of this choice stems from the existence of a zonal tilting of the thermocline, as noted in Constantin & Johnson [10]. With these aspects in mind we proceed to construct a family of exact solutions, presented in a rotating framework (by means of spherical coordinates), that represent incompressible azimuthal equatorial water flows with a free surface and a free interface. We also want to emphasize that the solutions we provide possess a depth-dependent velocity field. For studies concerning stratification, as well as the presence of an interface, in the equatorial scenario we refer the reader to [5, 11, 15, 27, 31, 34]. Concerning recent progress toward the understanding of several intricate features underlying the dynamics of coupled surface and internal waves we point out the excellent work by Henry & Villari [25].

The layout of this paper is as follows: after introducing the physical problem in Sect. 2 we derive in Sect. 3.1 formulas for the velocity field and the pressure in the two domains separated by the interface arising as a result of jump in the density. An implicit formula for the interface (which plays the role of an internal wave) is obtained in Sect. 3.2 by utilizing the balance of forces along it. Moreover, the dynamic boundary condition allows us to find a relation between the enforced pressure on the surface and the deviation of the surface from a surface following the Earth’s curvature. While the two implicit relations defining the free surface and the interface are quite involved they can be studied by means of the implicit function theorem. We conclude the paper with Sect. 4 where reasonable and expected physical properties of the exact solutions are presented. Indeed, it is proved that a growth in the pressure along the surface causes a decrease in the free surface height. Moreover, a regularity result concerning the interface defining function is also presented.

## Preliminaries

This section is concerned with the statement of the governing equations (together with their boundary conditions) for geophysical fluid dynamics (GFD). These are formulated in a spherical coordinate system which is fixed at a point on the Earth’s surface as will be detailed shortly. As a result the fine features of the Earth’s spherical geometry are reflected in the structure of our solutions. The latter are assumed to have a jet-like structure, capturing the observed azimuthal character of some equatorial flows. In the formulation of the governing equations we take into account the observation by Maslowe [33] that the Reynolds number is, in general, extremely large for the type of flows described above.

**Fig. 1:**
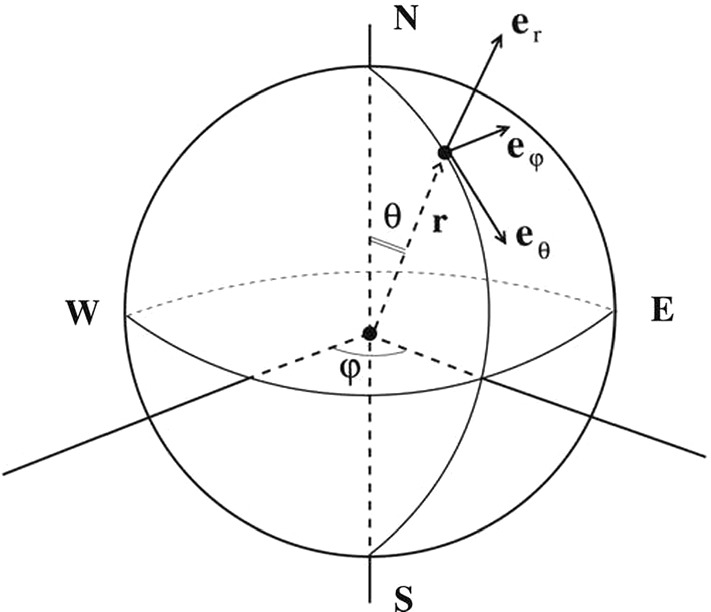
The spherical coordinate system as presented in Sect. 2. The variable *r* represents the distance from the origin, $\theta$ is the polar angle ($\pi /2-\theta$ being the angle of latitude), and $\varphi$ is the angle of longitude (azimuthal angle)

The right-handed coordinate system we will be using is denoted with $(r, \theta ,\varphi )$, where *r* is the distance from the centre of the earth, $\theta$ (with $0\le \theta \le \pi$) is the polar angle, and $\varphi$ (with $0\le \varphi < 2\pi$) is the azimuthal angle, see Fig. 1. The location of the North and South poles are at $\theta =0,\pi$, respectively, while the Equator is situated at $\theta =\frac{\pi }{2}$. The unit vectors in this $(r,\theta , \varphi )$ system are $(\mathbf {e}_r, \mathbf {e}_{\theta }, \mathbf {e}_{\varphi })$, respectively, and the corresponding velocity components are (*w*, *v*, *u*); $\mathbf {e}_{\varphi }$ points from West to East, and $\mathbf {e}_{\theta }$ points from North to South. More precisely, $\mathbf{e}_r=(\sin \theta \cos \varphi ,\sin \theta \sin \varphi , \cos \theta )$, $\mathbf{e}_{\theta }=(\cos \theta \cos \varphi ,\cos \theta \sin \varphi , -\sin \theta )$, $\mathbf{e}_{\varphi }=(-\sin \varphi ,\cos \varphi ,0).$

A noteworthy aspect is that the fluid domain is stratified where the stratification is brought about by the changes in the (discontinuous) density. To be more specific, denoting with $R\approx 6378$ km the Earth’s radius and with $r_0>0, r_1>0$ some constants, we assume that the flow consists of a upper layer

$$\begin{aligned} D_1:=\{(r,\theta , \varphi ): R_1+h(\theta , \varphi )\le r\le R_0+k(\theta ,\varphi )\}, \end{aligned}$$

of density $\rho _1(r,\theta )$ that lies above a bottom layer

$$\begin{aligned} D_2:=\{(r,\theta , \varphi ): R+d(\theta , \varphi )\le r\le R_1+h(\theta ,\varphi )\}, \end{aligned}$$

of density $\rho _2(r,\theta )$, with $R_0:=R+r_0$ and $R_1:=R+r_1$. While above $\theta \mapsto d(\theta )$ is a given function, $h(\theta , \varphi )$ and $k(\theta ,\varphi )$ are unknowns of the problem and denote the interface and the free surface defining functions, respectively.

### Remark 2.1

Based on data collected on physical grounds, it is appropriate to assume the relation $\rho _2=\rho _1(1+\sigma )$ where $\tau \mapsto \sigma (\tau )$ is a positive function with the additional property that $\sigma =\mathcal {O}(10^{-3})$.

Denoting by $\mathbf{u}=u\mathbf{e}_r+v\mathbf{e}_{\theta }+w\mathbf{e}_{\varphi }$ the velocity field we have that the governing equations in the rotating $(r, \theta , \varphi )$ coordinate system are the Euler’s equations,

1a $$\begin{aligned}&u_{i,t}+uu_{i,r} +\frac{v}{r}u_{i,\theta }+\frac{w}{r\sin \theta }u_{i,\varphi }-\frac{1}{r}(v_i^2+w_i^2) -2\Omega w_i\sin \theta -r\Omega ^2\sin ^2\theta \nonumber \\&\quad =-\frac{1}{\rho }p_r +F_r\nonumber \\&v_{i,t}+uv_{i,r} +\frac{v}{r}v_{i,\theta }+\frac{w}{r\sin \theta }v_{i,\varphi }+\frac{1}{r}(u_i v_i-w^2\cos \theta ) -2\Omega w_i\cos \theta -r\Omega ^2\sin \theta \cos \theta \nonumber \\&\quad = -\frac{1}{\rho }\frac{1}{r}p_{\theta } +F_{\theta }\nonumber \\&w_{i,t}+uw_{i,r} +\frac{v}{r}w_{\theta }+\frac{w}{r\sin \theta }w_{i,\varphi }+\frac{1}{r}(u_i w_i+v_i w_i\cot \theta ) +2\Omega (u_i\sin \theta +v_i\cos \theta )\nonumber \\&= -\frac{1}{\rho }\frac{1}{r\sin \theta }p_{\varphi } +F_{\varphi }, \end{aligned}$$

where $p(r,\theta ,\varphi )$ is denotes the pressure in the fluid and $(F_r,F_{\theta }, F_{\varphi })$ is the body-force vector, and the equation of mass conservation

1b $$\begin{aligned} \frac{1}{r^2}\frac{\partial }{\partial r}(\rho r^2 u_i)+\frac{1}{r\sin \theta }\frac{\partial }{\partial \theta }(\rho v_i\sin \theta )+\frac{1}{r\sin \theta }\frac{ \partial (\rho w_i)}{\partial \varphi }=0. \end{aligned}$$

The description of the water wave problem is completed by the specification of the associated boundary conditions. These are as follows. At the free-surface $r=R_0+k(\theta ,\varphi )$ we require the dynamic condition involving the surface pressure

2a $$\begin{aligned} p_1=P_1(\theta ,\varphi ), \end{aligned}$$

(for some given function $P_1(\theta ,\varphi )$) and the kinematic condition

2b $$\begin{aligned} w_1=\frac{v_1}{r}\frac{\partial k}{\partial \theta }+\frac{u_1}{r\sin \theta }\frac{\partial k}{\partial \varphi }, \end{aligned}$$

to hold.

At the interface, $r=R_1+h(\theta , \varphi )$, we ask that the normal components of the velocity fields from the upper and lower layer, respectively, are the same. The latter is equivalent with the condition

2c $$\begin{aligned} \begin{aligned} (w_1\mathbf {e}_r+v_1\mathbf {e}_{\theta }+u_1\mathbf {e}_{\varphi })&\cdot \left( \mathbf {e}_r-\frac{h_{\theta }}{r}\mathbf {e}_{\theta }-\frac{h_{\varphi }}{r\sin \theta }\mathbf {e}_{\varphi }\right) \\&=(w_2\mathbf {e}_r+v_2\mathbf {e}_{\theta }+u_2\mathbf {e}_{\varphi })\cdot \left( \mathbf {e}_r-\frac{h_{\theta }}{r}\mathbf {e}_{\theta }-\frac{h_{\varphi }}{r\sin \theta }\mathbf {e}_{\varphi }\right) . \end{aligned} \end{aligned}$$

Moreover, to ensure the balance of forces at the interface, we also require that the pressure from the upper layer coincides with the pressure from the bottom layer, that is,

2d $$\begin{aligned} p_1(R+h(\theta ,\varphi ),\theta ,\varphi )=p_2(R+h(\theta ,\varphi ), \theta ,\varphi ). \end{aligned}$$

At the bottom of the ocean, which is an impermeable, solid boundary described by the equation $r=d(\theta ,\varphi )$, the associated kinematic condition is

2e $$\begin{aligned} w_2=\frac{v_2}{r}\frac{\partial d}{\partial \theta }+\frac{u_2}{r\sin \theta }\frac{\partial d}{\partial \varphi }. \end{aligned}$$

## Explicit solutions

We aim at finding solutions of the Eqs. (1a), (1b) and (2) that represent purely-azimuthal steady flows with no variation in the azimuthal direction. That is to say that the velocity field satisfies $u_1=v_1=u_2=v_2=0$ and $w_1=w_1(r,\theta ),\,w_2=w_2(r,\theta )$. Moreover, the other unknows of the problem are characterized by the properties $p_1=p_1(r,\theta ),\,p_2=p_2(r,\theta ),\,h=h(\theta ),\,k=k(\theta )$. Throughout the section the range for the $\theta$ variable will be the interval $\left[ \frac{\pi }{2},\frac{\pi }{2}+\varepsilon \right]$ where $\varepsilon =0.016$ determines a strip of about 100 km about the Equator.

### The velocity and the pressure

We notice that a flow with the previous features automatically satisfies the boundary and interface conditions (2) as well as the equation of mass conservation (1b). Assuming that the only body-force is due to gravity alone, that is the body-force vector is $-g\mathbf {e}_r$, the Euler equations are written as

3 $$\begin{aligned} \left\{ \begin{array}{rcl} -\frac{w_i^2}{r}-2\varOmega w_i\sin \theta -r\varOmega ^2\sin ^2 \theta &{} =&{} -\frac{1}{\rho _i}p_{i,r}-g\\ &{} &{}\\ -\frac{ w_i^2}{r}\cot \theta - 2\varOmega w_i\cos \theta -r\varOmega ^2\sin \theta \cos \theta &{} = &{} -\frac{1}{\rho _i r}p_{i,\theta }\\ &{} &{}\\ 0&{} =&{} -\frac{1}{\rho _i}\frac{1}{r\sin \theta }p_{i,\varphi } \end{array},\right. \end{aligned}$$

in the domain $D_i$ for $i=1,2$. In the first step we simplify the system above by eliminating the pressue. So we obtain that the azimuthal components of the velocity field $w_i$ satisfy

4 $$\begin{aligned} \partial _{\theta }\left( \frac{\rho (r)( w_i(r,\theta )+\Omega r\sin \theta )^2}{r}\right) -\partial _r\left( \rho (r)( w_i(r,\theta )+\Omega r\sin \theta )^2\cot \theta \right) =0, \end{aligned}$$

for $i=1,2$. Utilizing the method of characteristics and adapting the approach from [23] we obtain that the azimuthal velocities $w_i (i=1,2)$ are expressed in terms of the formulas

5 $$\begin{aligned} w_i(r,\theta )=-\Omega r\sin \theta +\sqrt{ \frac{ F_i^2(r\sin \theta )+ g r\sin \theta \int _0^{f(\theta )}\left[ \rho _{i,\theta }(\overline{r}(s),\overline{\theta }(s)) \right] ds }{\rho _i(r,\theta ) } }, \end{aligned}$$

where $x\rightarrow F_i(x)$, $i=1,2$, are some real-valued functions, while

6 $$\begin{aligned} \begin{aligned} f(\theta )&=\ln \left( \sqrt{\frac{1-\cos \theta }{1+\cos \theta }}\right) ,\\ \overline{r}(s)&=r\sin \theta \cosh (s),\\ \overline{\theta }(s)&=\arccos (-\sinh (s)). \end{aligned} \end{aligned}$$

Toward deriving the formula for the pressure we first infer from (3) that the pressure gradient is given as

7 $$\begin{aligned} p_{i,r}= \frac{ F_i^2(r\sin \theta )+ g r\sin \theta \int _0^{f(\theta )}\left[ \rho _{i,\theta }(\overline{r}(s),\overline{\theta }(s)) \right] ds }{ r } -g\rho _i \end{aligned}$$

and

8 $$\begin{aligned} p_{i,\theta } =\cot \theta \left[ F_i^2(r\sin \theta )+ r\sin \theta \int _0^{f(\theta )}\left[ g\rho _{i,\theta }(\overline{r}(s),\overline{\theta }(s)) \right] ds \right] \end{aligned}$$

for $i=1,2$. An integration with respect to *r* yields that for all $r\in [R+d(\theta ), R_1+h(\theta )]$ we have

9 $$\begin{aligned} p_2(r,\theta )=\int _{(R+d(\theta ))\sin \theta }^{r\sin \theta }\left( \frac{F_2^2(y)}{y} +\mathcal {F}_2(y,\theta )\right) dy -g\int _{R+d(\theta )}^r\rho _2(\tilde{r},\theta )d\tilde{r} +C_2(\theta ), \end{aligned}$$

where

$$\begin{aligned} \mathcal {F}_2(y,\theta ):=g\int _0^{f(\theta )}\rho _{2,\theta }\left( y\cdot \cosh (s),\overline{\theta }(s)\right) ds, \end{aligned}$$

and $\theta \rightarrow C_2(\theta )$ is a function such that

10 $$\begin{aligned} \begin{aligned} C_2'(\theta )=&\frac{F_2^2((R+d(\theta ))\sin \theta )}{ ((R+d(\theta ))\sin \theta )}\cdot \frac{d}{d\theta }((R+d(\theta ))\sin \theta )\\&+\mathcal {F}((R+d(\theta ))\sin \theta )\frac{d}{d\theta }((R+d(\theta ))\sin \theta )-g\rho _2(R+d(\theta ),\theta )d'(\theta ). \end{aligned} \end{aligned}$$

We proceed now with the determination of the pressure in the upper layer $D_1$. We recall that the interface (which is the lower boundary of $D_1$) is given as $r=R_1+h(\theta )$. An integration with respect to *r* in formula (7) yields that for all $\theta$ and all $r\in [R_1+h(\theta ), R_0+k(\theta )]$ it holds

11 $$\begin{aligned} p_1(r,\theta )=\int _{(R_1+h(\theta ))\sin \theta }^{r\sin \theta }\left( \frac{F_1^2(y)}{y} +\mathcal {F}_1(y,\theta )\right) dy -g\int _{R_1+h(\theta )}^r\rho _1(\tilde{r},\theta )d\tilde{r}+C_1(h,\theta ),\nonumber \\ \end{aligned}$$

where

12 $$\begin{aligned} \mathcal {F}_1(y,\theta ):=g\int _0^{f(\theta )}\rho _{1,\theta }\left( y\cdot \cosh (s),\overline{\theta }(s)\right) ds, \end{aligned}$$

and

13 $$\begin{aligned} \begin{aligned} C_1(h,\theta )=&\int _{\pi /2}^{\theta }F_1^2\big ((R_1+h(\tilde{\theta }))\sin \tilde{\theta }\big )\left[ \cot \tilde{\theta }+ \frac{h'(\tilde{\theta })}{R_1+h(\tilde{\theta })}\right] \,d\tilde{\theta }\\&+\int _{\pi /2}^{\theta }\mathcal {F}_1\big ((R_1+h(\tilde{\theta }))\sin \tilde{\theta },\tilde{\theta }\big ) \left[ h'(\tilde{\theta }) \sin \tilde{\theta } +(R_1+h(\tilde{\theta }))\cos \tilde{\theta }\right] \,d\tilde{\theta }\\&-g\int _{\pi /2}^{\theta }\rho _1(R_1+h(\tilde{\theta }), \tilde{\theta })h'(\tilde{\theta })\,d\tilde{\theta }. \end{aligned}\nonumber \\ \end{aligned}$$

### The interface and the free surface

Having the velocity and the pressure determined we pass now to the (implicit) determination of the two interface defining functions *h* and *k*. To this end we first exploit the balance of forces at the interface (2d), written now (in the context of azimuthal flows) as

14 $$\begin{aligned} p_1(R_1+h(\theta ),\theta )=p_2(R_1+h(\theta ),\theta ), \end{aligned}$$

which can be detailed as

15 $$\begin{aligned} C_1(h,\theta )=\!\int _{(R+d(\theta ))\sin \theta }^{(R_1+h(\theta ))\sin \theta }\!\left( \frac{F_2^2(y)}{y} +\mathcal {F}_2(y,\theta )\!\right) dy -g\int _{R+d(\theta )}^{R_1+h(\theta )}\!\rho _2(\tilde{r},\theta )d\tilde{r}+C_2(\theta ).\nonumber \\ \end{aligned}$$

We provide now a functional analytic setting for our problem by nondimensionalizing the interface defining function. That is, we set

$$\begin{aligned} \mathcal {h}(\theta ):=\frac{h(\theta )}{R_1} \end{aligned}$$

and so write (14) as

$$\begin{aligned} \mathcal {G}_2(\mathcal {h})=0 \end{aligned}$$

where the operator $\mathcal {G}_2$ acts from the Banach space $C^1\left( \left[ \frac{\pi }{2},\frac{\pi }{2}+\varepsilon \right] \right)$ into itself and is given as

16 $$\begin{aligned} \begin{aligned} \mathcal {G}_2(\mathcal {h})=&\frac{1}{P_{atm}}\left( \int _{(R+d(\theta ))\sin \theta }^{(1+\mathcal {h}(\theta ))R_1\sin \theta }\!\left( \frac{F_2^2(y)}{y} +\mathcal {F}_2(y,\theta )\!\right) dy -g\int _{R+d(\theta )}^{(1+\mathcal {h}(\theta ))R_1}\!\rho _2(\tilde{r},\theta )d\tilde{r}\right) \\&\frac{-C_1(\mathcal {h},\theta )+C_2(\theta )}{P_{atm}}. \end{aligned}\nonumber \\ \end{aligned}$$

To recover the shape of the free surface (at least in an implicite form) we use the dynamic boundary condition (2a) and so obtain that a prescribed pressure at the surface $P_1(\theta )$ has to satisfy the equation

17 $$\begin{aligned} P_1(\theta )= \int _{(R_1+h(\theta ))\sin \theta }^{(R_0+k(\theta ))\sin \theta }\left( \frac{F_1^2(y)}{y} +\mathcal {F}_1(y,\theta )\right) dy -g\int _{R_1+h(\theta )}^{R_0+k(\theta )}\rho _1(\tilde{r},\theta )d\tilde{r}+C_1(h,\theta ),\nonumber \\ \end{aligned}$$

called the Bernoulli relation which establishes a connection between the pressure applied on the free surface on one hand and, on the othe hand, the shape of the free surface and the shape of the interface, where the latter is determined by Eq. (15). Setting

$$\begin{aligned} \mathcal {k}(\theta ):=\frac{k(\theta )}{R_0},\,\,\mathcal {P}_1(\theta ):=\frac{P_1(\theta )}{P_{atm}} \end{aligned}$$

we recast the previous equation in nondimensional form as the abstract operatorial equation

18 $$\begin{aligned} \mathcal {G}_1(\mathcal {k},\mathcal {h}, \mathcal {P}_1)=0, \end{aligned}$$

where $\mathcal {G}_1$ is an operator from the Banach space $C\left( \left[ \frac{\pi }{2},\frac{\pi }{2}+\varepsilon \right] \right) \times C^1\left( \left[ \frac{\pi }{2},\frac{\pi }{2}+\varepsilon \right] \right) \times C\left( \left[ \frac{\pi }{2},\frac{\pi }{2}+\varepsilon \right] \right)$ into itself and is given through

19 $$\begin{aligned} \begin{aligned}&\mathcal {G}_1 (\mathcal {k},\mathcal {h},\mathcal {P}_1)(\theta )= \frac{1}{P_{atm}}\left( \int _{(1+\mathcal {h}(\theta ))R_1\sin \theta }^{(1+\mathcal {k}(\theta ))R_0\sin \theta }\left( \frac{F_1^2(y)}{y} +\mathcal {F}_1(y,\theta )\right) dy\right. \\&\quad \left. -g\int _{(1+\mathcal {h}(\theta ))R_1}^{(1+\mathcal {k}(\theta ))R_0}\rho _1(\tilde{r},\theta )d\tilde{r}+C_1(\mathcal {h},\theta )\right) ,-\mathcal {P}_1(\theta ). \end{aligned} \end{aligned}$$

The problem of finding $(\mathcal {k},\mathcal {h})$ that satisfy (15) and (17) can be written now as

20 $$\begin{aligned} ( \mathcal {G}_1(\mathcal {k},\mathcal {h}, \mathcal {P}_1), \mathcal {G}_2(\mathcal {h}))=0. \end{aligned}$$

In order to prove the existence of nontrivial solutions $\mathcal {h},\mathcal {k}$ to (20) we are going to utilize the Implicit Function Theorem, cf. [1]. In order to do so, we need first to identify trivial solutions $(\mathcal {k}_0,\mathcal {h}_0)$ of (20). The natural candidate for a trivial solution is the flow having an undisturbed free surface and an undisturbed interface following the Earth’s curvature. That is, we set $\mathcal {k}=\mathcal {h}=0$ in (20) and find that $\mathcal {G}_1(\mathcal {0},\mathcal {0}, \mathcal {P}_1^0)=\mathcal {G}_2(\mathcal {0})=0$ if and only if

21 $$\begin{aligned} \begin{aligned} \mathcal {P}_1^0=&\frac{1}{P_{atm}}\left( \int _{R_1\sin \theta }^{R_0\sin \theta } \left( \frac{F_1^2(y)}{y} +\mathcal {F}_1(y,\theta ) \right) dy-g\int _{R_1}^{R_0}\rho _1(\tilde{r},\theta )\,d\tilde{r}\right) \\&+\frac{1}{P_{atm}}\left( \int _{\pi /2}^{\theta }F_1^2(R_1\sin \tilde{\theta })\cot \tilde{\theta }\,d\tilde{\theta } +\int _{\pi /2}^{\theta }\mathcal {F}_1(R_1\sin \tilde{\theta },\tilde{\theta })R_1\cos \tilde{\theta }\,d\tilde{\theta }\right) \end{aligned} \end{aligned}$$

and

22 $$\begin{aligned} \begin{aligned}&\frac{1}{P_{atm}} \!\int _{(R+d(\theta ))\sin \theta }^{R_1\sin \theta }\!\left( \frac{F_2^2(y)}{y} +\mathcal {F}_2(y,\theta )\!\right) dy -g\int _{R+d(\theta )}^{R_1}\!\rho _2(\tilde{r},\theta )d\tilde{r} +C_2(\theta ) \\&\quad -\frac{1}{P_{atm}}\left( \int _{\pi /2}^{\theta }F_1^2(R_1\sin \tilde{\theta })\cot \tilde{\theta }\,d\tilde{\theta } +\int _{\pi /2}^{\theta }\mathcal {F}_1(R_1\sin \tilde{\theta },\tilde{\theta })R_1\cos \tilde{\theta }\,d\tilde{\theta }\right) =0. \end{aligned} \end{aligned}$$

We evaluate

23 $$\begin{aligned} \lim _{s\rightarrow 0}\frac{C_1(sh,\theta )-C_1(0,\theta )}{s}. \end{aligned}$$

To begin with we compute

24 $$\begin{aligned} \begin{aligned}&\lim _{s\rightarrow 0}\frac{1}{s}\int _{\pi /2}^{\theta }\mathcal {F}_1\left( R_1+s h(\tilde{\theta }))\sin \tilde{\theta },\tilde{\theta }\right) \left[ sh'(\tilde{\theta })\sin \tilde{\theta }+sh(\tilde{\theta })\cos \tilde{\theta }\right] d\tilde{\theta }\\&\quad \quad +\lim _{s\rightarrow 0}\int _{\pi /2}^{\theta } \frac{ \mathcal {F}_1\left( R_1+s h(\tilde{\theta }))\sin \tilde{\theta },\tilde{\theta }\right) -\mathcal {F}_1\left( R_1\sin \tilde{\theta },\tilde{\theta }\right) }{s} R_1\cos \tilde{\theta }d\tilde{\theta }\\&\quad =\int _{\pi /2}^{\theta }\mathcal {F}_1(R_1\sin \tilde{\theta },\tilde{\theta })\frac{d}{d\tilde{\theta }}(h(\tilde{\theta })\sin \tilde{\theta })d\tilde{\theta }+\int _{\pi /2}^{\theta }\mathcal {F}_{1,y}(R_1\sin \tilde{\theta },\tilde{\theta }) R_1 h(\tilde{\theta })\sin \tilde{\theta }\cos \tilde{\theta }d\tilde{\theta }\\&\quad =\mathcal {F}_1(R_1\sin \theta ,\theta ) h(\theta )\sin \theta )-\int _{\pi /2}^{\theta }\mathcal {F}_{1,\theta } (R_1\sin \tilde{\theta },\tilde{\theta })(h(\tilde{\theta })\sin \tilde{\theta })d\tilde{\theta }\\&\quad =\mathcal {F}_1(R_1\sin \theta ,\theta ) h(\theta )\sin \theta -g\int _{\pi /2}^{\theta }\rho _{1,\theta }(R_1,\tilde{\theta })h(\tilde{\theta })d\tilde{\theta } \end{aligned}\nonumber \\ \end{aligned}$$

Moreover,

25 $$\begin{aligned} \begin{aligned}&\lim _{s\rightarrow 0}\int _{\pi /2}^{\theta }F_1^2\left( (R_1+s h(\tilde{\theta }))\sin \tilde{\theta }\right) \frac{h'(\tilde{\theta })d\tilde{\theta }}{R_1+s h(\tilde{\theta })}\\&\quad \lim _{s\rightarrow 0}\int _{\pi /2}^{\theta } \frac{F_1^2\left( (R_1+s h(\tilde{\theta }))\sin \tilde{\theta }\right) -F_1^2\left( R_1\sin \tilde{\theta }\right) }{s}\cot \tilde{\theta }d\tilde{\theta }\\&\quad =F_1^2(R_1\sin \theta )\frac{h(\theta )}{R_1}-\int _{\pi /2}^{\theta }(F_1^2)'(R_1\sin \tilde{\theta })R_1\cos \tilde{\theta } \frac{h(\tilde{\theta })}{R_1}d\tilde{\theta }\\&\qquad +\lim _{s\rightarrow 0}\int _{\pi /2}^{\theta }\frac{s h(\tilde{\theta })\sin \tilde{\theta }}{s}(F_1^2)'(R_1\sin \tilde{\theta })\cot \tilde{\theta }d\tilde{\theta }\\&\quad =F_1^2(R_1\sin \theta )\frac{h(\theta )}{R_1} \end{aligned} \end{aligned}$$

and

26 $$\begin{aligned} \begin{aligned}&\lim _{s\rightarrow 0}\int _{\pi /2}^{\theta } \frac{\rho _1\left( R_1+sh(\tilde{\theta }),\tilde{\theta }\right) sh'(\tilde{\theta })}{s}d\tilde{\theta }\\&\quad =\int _{\pi /2}^{\theta }\rho _1(R_1,\tilde{\theta }) h'(\tilde{\theta })d\tilde{\theta } =\rho _1(R_1,\theta )h(\theta )-\int _{\pi /2}^{\theta }\rho _{1,\theta }(R_1,\tilde{\theta })h(\tilde{\theta })d\tilde{\theta }. \end{aligned} \end{aligned}$$

Collecting now (24),(25) and (26) and recalling formula (13) we have

27 $$\begin{aligned} \begin{aligned} \lim _{s\rightarrow 0}\frac{C_1(sh,\theta )-C_1(0,\theta )}{s}=&\mathcal {F}_1(R_1\sin \theta ,\theta ) h(\theta )\sin \theta + F_1^2(R_1\sin \theta )\frac{h(\theta )}{R_1}\\&-g\rho _1(R_1,\theta )h(\theta ). \end{aligned} \end{aligned}$$

Moreover

28 $$\begin{aligned} \begin{aligned}&\int _{(1+s\mathcal {h}(\theta ))R_1\sin \theta }^{(1+\mathcal {k}(\theta ))R_0\sin \theta }\left( \frac{F_1^2(y)}{y} +\mathcal {F}_1(y,\theta )\right) dy- \int _{R_1\sin \theta }^{(1+\mathcal {k}(\theta ))R_0\sin \theta }\left( \frac{F_1^2(y)}{y} +\mathcal {F}_1(y,\theta )\right) dy\\&\quad = \int _{(1+s\mathcal {h}(\theta ))R_1\sin \theta }^{R_1\sin \theta }\left( \frac{F_1^2(y)}{y} +\mathcal {F}_1(y,\theta )\right) dy \end{aligned} \end{aligned}$$

Appealing to the mean value theorem for integrals we obtain

29 $$\begin{aligned} \begin{aligned}&\lim _{s\rightarrow 0}\frac{1}{s}\int _{(1+s\mathcal {h}(\theta ))R_1\sin \theta }^{R_1\sin \theta }&\left( \frac{F_1^2(y)}{y} +\mathcal {F}_1(y,\theta )\right) dy\\&\quad =-\mathcal {F}_1(R_1\sin \theta ,\theta ) h(\theta )\sin \theta - F_1^2(R_1\sin \theta )\frac{h(\theta )}{R_1} \end{aligned} \end{aligned}$$

and

30 $$\begin{aligned} \begin{aligned}&\lim _{s\rightarrow 0} \frac{1}{s}\left( \int _{(1+s\mathcal {h}(\theta ))R_1}^{(1+\mathcal {k}(\theta ))R_0}\rho _1(\tilde{r},\theta )d\tilde{r}-\int _{R_1}^{(1+\mathcal {k}(\theta ))R_0}\rho _1(\tilde{r},\theta )d\tilde{r}\right) \\&\quad =\lim _{s\rightarrow 0}\frac{1}{s}\int _{(1+s\mathcal {h}(\theta ))R_1}^{R_1}\rho _1(\tilde{r},\theta )d\tilde{r}=\rho _1(R_1,\theta )R_1\mathcal {h}(\theta )=\rho _1(R_1,\theta )h(\theta ) \end{aligned} \end{aligned}$$

Therefore, taking into account formulas (27),(29),(30) as well as the definition of the operator $\mathcal {G}_1$ in (19) we obtain

31 $$\begin{aligned} \mathcal {G}_{1\mathcal {h}}(0,0,\mathcal {P}^0_1)\mathcal {h}=\lim _{s\rightarrow 0}\frac{\mathcal {G}_1(0,s\mathcal {h},\mathcal {P}^0_1 )-\mathcal {G}_1(0,0,\mathcal {P}^0_1 )}{s}=0 \end{aligned}$$

Similarly, we compute

32 $$\begin{aligned} \begin{aligned} \!\!\!\!\!\left( \mathcal {G}_{1\mathcal {k}}(0,0,\mathcal {P}_1^0)\mathcal {k}\right) (\theta )&=\lim _{s\rightarrow 0}\frac{\mathcal {G}_1(s\mathcal {k},0,\mathcal {P}^0_1 )(\theta )-\mathcal {G}_1(0,0,\mathcal {P}^0_1 )(\theta )}{s}\\&=\frac{(F_1^2(R_0\sin \theta )+\mathcal {F}_1(R_0\sin \theta ,\theta )R_0\sin \theta -gR_0\rho _1(R_0,\theta ))\mathcal {k}(\theta )}{P_{atm}}\\&=\frac{\rho _1(R_0,\theta )}{P_{atm}}[w(R_0,\theta )+\Omega R_0\sin \theta )^2-gR_0]\mathcal {k}(\theta ). \end{aligned}\nonumber \\ \end{aligned}$$

Performing calculations analogous to (29) we infer that

33 $$\begin{aligned} \begin{aligned}&\left( \mathcal {G}_{2\mathcal {h}}(0)\mathcal {h}\right) (\theta )\\&\quad = \lim _{s\rightarrow 0}\frac{\mathcal {G}_2(s\mathcal {h})(\theta )-\mathcal {G}_2(0)(\theta ) }{s}\\&\quad =\frac{\left( F_2^2(R_1\sin \theta )+\mathcal {F}_2(R_1\sin \theta ,\theta )R_1\sin \theta \right) \mathcal {h}(\theta )}{P_{atm}}\\&\qquad -\frac{\left( F_1^2(R_1\sin \theta )+\mathcal {F}_1(R_1\sin \theta ,\theta )R_1\sin \theta \right) \mathcal {h}(\theta )}{P_{atm}}\\&\qquad -\frac{g R_1(\rho _2(R_1,\theta )-\rho _1(R_1,\theta ))\mathcal {h}(\theta )}{P_{atm}}\\&\quad =\frac{(w_2(R_1,\theta )+\Omega R_1\sin \theta )^2\rho _2(R_1,\theta )-(w_1(R_1,\theta )+\Omega R_1\sin \theta )^2\rho _1(R_1,\theta ))}{P_{atm}}\mathcal {h}(\theta )\\&\qquad -\frac{g R_1(\rho _2(R_1,\theta )-\rho _1(R_1,\theta ))}{P_{atm}} \mathcal {h}(\theta ). \end{aligned} \end{aligned}$$

If we are to consider $\mathcal {G}_2$ as an operator of $\mathcal {k}$, $\mathcal {h}$ and $\mathcal {P}$ then clearly, $\mathcal {G}_{2\mathcal {k}}(0,0,\mathcal {P}_1^0)\mathcal {k}=0$ for all $\mathcal {k}$. Therefore,34as a linear operator from $C\big ([\pi /2,\pi /2+\varepsilon ]\big )\times C^1\big ([\pi /2,\pi /2+\varepsilon ]\big )$ into itself.

#### Theorem 3.1

Any sufficiently small perturbation $\mathcal {P}$ of $\mathcal {P}^0_1$ gives rise to a unique tuple $(\mathcal {k},\mathcal {h})\in C\big ([\pi /2,\pi /2+\varepsilon ]\big )\times C^1\big ([\pi /2,\pi /2+\varepsilon ]\big )$ which satisfies the equation $(\mathcal {G}_1(\mathcal {k},\mathcal {h},\mathcal {P}_1),\mathcal {G}_2(\mathcal {h}))=0$.

#### Proof

To begin with we note that, due to Remark 2.1, we can write

35 $$\begin{aligned} \begin{aligned}&(w_2(R_1,\theta )+\Omega R_1\sin \theta )^2\rho _2(R_1,\theta )-(w_1(R_1,\theta )+\Omega R_1\sin \theta )^2\rho _1(R_1,\theta ))\\&\quad =\rho _1(R_1,\theta )(w_2^2-w_1^2 +2\Omega R_1(w_2-w_1)\sin \theta ) +\rho _1\sigma (w_2(R_1,\theta )+\Omega R_1\sin \theta )^2\\&\quad<\rho _1(R_1,\theta )\Big ( w_2 (R_1,\theta )[w_2(R_1,\theta )+2\Omega R_1\sin \theta ]+\sigma (w_2(R_1,\theta )+\Omega R_1\sin \theta )^2\big )\\&\quad < R_1\,kg\cdot m^{-2}\cdot s^{-2}. \end{aligned}\nonumber \\ \end{aligned}$$

On the other hand

36 $$\begin{aligned} gR_1(\rho _2(R_1,\theta )-\rho _1(R_1,\theta ))=gR_1\rho _1\sigma > 4.9 R_1\,kg\cdot m^{-2}\cdot s^{-2} \end{aligned}$$

From (33), (35) and (36) we conclude that there is a negative constant $\mathcal {a}$ such that $(\mathcal {G}_{2\mathcal {h}}(0,0,\mathcal {P}_1^0)\mathcal {h})(\theta )\le \mathcal {a}$ for all $\theta \in [0,\pi ]$. Consequently, the map

37 $$\begin{aligned} \begin{aligned} C^1\big ([\pi /2,\pi /2+\varepsilon ]\big )&\rightarrow C^1\big ([\pi /2,\pi /2+\varepsilon ]\big ),\\ \mathcal {h}&\mapsto \mathcal {G}_{2\mathcal {h}}(0,0,\mathcal {P}_1^0)\mathcal {h} \end{aligned} \end{aligned}$$

is a linear homeomorphism. On the other hand, since the typical values for velocity in the ocean do not exceed 4 m$\cdot s^{-1}$ we see that $\big (w(R_0,\theta )+\Omega R_0\sin \theta )\big )^2$ is much smaller than $gR_0$. Hence, we can conclude that there exists $\mathcal {b}<0$ such that $(\mathcal {G}_{1\mathcal {k}}(0,0,\mathcal {P}_1^0)\mathcal {k})(\theta )\le \mathcal {b}$ for all $\theta \in [0,\pi ]$. Thus, the map

38 $$\begin{aligned} \begin{aligned} C\big ([\pi /2,\pi /2+\varepsilon ]\big )&\rightarrow C\big ([\pi /2,\pi /2+\varepsilon ]\big ),\\ \mathcal {k}&\mapsto \mathcal {G}_{1\mathcal {k}}(0,0,\mathcal {P}_1^0)\mathcal {k} \end{aligned} \end{aligned}$$

is a linear homeomorphism. In view of (34), (37) and (38) we can now conclude that  is homeomorphism from $C\big ([\pi /2,\pi /2+\varepsilon ]\big )\times C^1\big ([\pi /2,\pi /2+\varepsilon ]\big )$ into itself. Resorting now to the implicit function theorem [1] delivers the assertion made in the statement of the theorem. $\square$

## Properties of the exact solutions

This section is devoted to the analysis of the exact solutions found in Sect. 3.2. The first result indicates that our solutions in spherical coordinates present expected physical properties: while a growth in pressure on the surface leads to a decrease in the free surface height, the increase in the latter brings about a attenuation of the former.

### Remark 4.1

For the next result we will assume that the given pressure $\mathcal {P}_1$ on the free surface is a differentiable function of $\theta$. Then an iterative bootstrapping procedure, cf. [1], ensures the differentiability of the surface defining function $\mathcal {k}$.

### Theorem 4.2

Denoting, as before, with $\mathcal {k}$ the function defining the free surface and with $\mathcal {P}_1$ the pressure on the latter, we have that

39 $$\begin{aligned} \mathcal {P}_1'(\theta )<0\quad \text {if}\quad \mathcal {k}'(\theta )\ge 0, \end{aligned}$$

and

40 $$\begin{aligned} \mathcal {k}'(\theta )<0\quad \text {if}\quad \mathcal {P}_1'(\theta )\ge 0, \end{aligned}$$

for all $$\theta \in \left( \frac{\pi }{2},\frac{\pi }{2}+\varepsilon \right) $$.

### Proof

We differentiate with respect to $\theta$ in equation (18) and obtain

41 $$\begin{aligned} \begin{aligned}&P_{atm}\mathcal {P}^{'}_1(\theta )\\&\quad = \left[ \frac{F_1^2\big ((1+\mathcal {k}(\theta ))R_0\sin \theta \big )}{1+\mathcal {k}(\theta )}+(R_0\sin \theta )\mathcal {F}_1\big ((1+\mathcal {k}(\theta ))R_0\sin \theta ,\theta \big )\right] \mathcal {k}^{'}(\theta )\\&\qquad -g\rho _1\big ((1+\mathcal {k}(\theta ))R_0,\theta \big )R_0\mathcal {k}^{'}(\theta )\\&\qquad +\left[ F_1^2\big ((1+\mathcal {k}(\theta ))R_0\sin \theta \big ) +\big (1+\mathcal {k}(\theta ))R_0\sin \theta \mathcal {F}_1\big ((1+\mathcal {k}(\theta ))R_0\sin \theta ,\theta \big )\right] \cot \theta \\&\quad =\Big [\frac{\Big (w_1(R_0(1+\mathcal {k}(\theta )),\theta )+\big (\Omega R_0(1+\mathcal {k}(\theta ))\sin \theta \Big )^2}{1+\mathcal {k}(\theta )} -gR_0\Big ] \rho _1(R_0(1+\mathcal {k}(\theta )),\theta )\mathcal {k}^{'}(\theta )\\&\qquad +\Big (w_1(R_0(1+\mathcal {k}(\theta )),\theta )+\big (\Omega R_0(1+\mathcal {k}(\theta ))\sin \theta \Big )^2 \rho _1(R_0(1+\mathcal {k}(\theta )),\theta )\cot \theta \end{aligned} \end{aligned}$$

where the last equality follows via (5) and (12). The conclusion in the statement of the theorem emerges now by noticing that

$$\begin{aligned} \frac{\Big (w_1(R_0(1+\mathcal {k}(\theta )),\theta )+\big (\Omega R_0(1+\mathcal {k}(\theta ))\sin \theta \Big )^2}{1+\mathcal {k}(\theta )} -gR_0<0 \end{aligned}$$

for values of $w_1$ that bear relevance on physical grounds. $\square$

### Theorem 4.3

Let us assume that between the two densities $\rho _1$ and $\rho _2$ we have the relation

42 $$\begin{aligned} \rho _2=\rho _1(1+\sigma ), \end{aligned}$$

where $\tau \mapsto \sigma (\tau )$ is a positive function so that $\sigma =\mathcal {O}(10^{-3})$, cf. [28]. Then the interface defining function $\theta \mapsto \mathcal {h}(\theta )$ has one degree of smoothness more than the velocity field. That is, if $F_1$ and $F_2$ are *k*-times differentiable, then $\mathcal {h}\in C^{k+1}\left[ \frac{\pi }{2},\frac{\pi }{2}+\varepsilon \right]$. Moreover, if $F_1$ and $F_2$ are infinitely many times differentiable, then $\mathcal {h}\in C^{\infty }\left[ \frac{\pi }{2},\frac{\pi }{2}+\varepsilon \right]$.

### Proof

Differentiating with respect to $\theta$ in (14) we obtain the equation

43 $$\begin{aligned} A(\theta )\mathcal {h}^{'}(\theta )+B(\theta )=0, \end{aligned}$$

where

44 $$\begin{aligned} \begin{aligned} A(\theta )=&\frac{\big (w_2(R_1+R_1\mathcal {h},\theta )+\Omega R_1(1+\mathcal {h})\sin \theta )\big )^2}{1+\mathcal {h}}\rho _2(R_1+R_1\mathcal {h})\\&-\frac{\big (w_1(R_1+R_1\mathcal {h},\theta )+\Omega R_1(1+\mathcal {h})\sin \theta )\big )^2}{1+\mathcal {h}}\rho _1(R_1+R_1\mathcal {h})\\&-gR_1\big (\rho _2(R_1+R_1\mathcal {h},\theta )-\rho _1(R_1+R_1\mathcal {h},\theta )\big ) \end{aligned} \end{aligned}$$

and

45 $$\begin{aligned} \begin{aligned} B(\theta )=&\left[ F_2^2\big ((1+\mathcal {h})R_1\sin \theta \big )+(1+\mathcal {h})R_1\sin \theta \mathcal {F}_2\big ((1+\mathcal {h})R_1\sin \theta \big )\right] \cot \theta \\&-\left[ F_1^2\big ((1+\mathcal {h})R_1\sin \theta \big )+(1+\mathcal {h})R_1\sin \theta \mathcal {F}_1\big ((1+\mathcal {h})R_1\sin \theta \big )\right] \cot \theta . \end{aligned} \end{aligned}$$

The last part of the proof consists in showing that $A(\theta )<0$ for all $\theta \in \left[ \frac{\pi }{2},\frac{\pi }{2}+\epsilon \right]$. To this end, we note that assumption (42) yields46The expression in the bracket above is negative as we can deduce by taking into account the size of the physical quantities involved. Hence $A(\theta )$ is negative for all $\theta \in \left[ \frac{\pi }{2},\frac{\pi }{2}+\epsilon \right]$. An iterative argument shows now the asserted differentiability of $\mathcal {h}$. $\square$
